# A Case of Elevated Troponin I Level After Packed Red Blood Cell Transfusion With Normal Coronary Angiography

**DOI:** 10.7759/cureus.26193

**Published:** 2022-06-22

**Authors:** Sherif Roman, Manpreet Sran, Amgad N Makaryus

**Affiliations:** 1 Internal Medicine, St. Joseph's Regional Medical Center, Paterson, USA; 2 Internal Medicine, Infinity Hospice Care, Las Vegas, USA; 3 Cardiology, Northwell Health, Manhasset, USA; 4 Cardiology, Nassau University Medical Center, East Meadow, USA

**Keywords:** microparticle enzyme immunoassay, fluorometric immunoassay, chemiluminescent immunoassay, acute coronary syndrome, laboratory testing, troponin

## Abstract

Other than acute coronary syndrome (ACS), many clinical conditions are associated with increased cardiac troponin I (cTnI) levels. Conditions such as pulmonary embolism, acute heart failure, myocarditis, sepsis, and renal failure are commonly reported as underlying causes. Analytical interference with the cTnI assay can also lead to falsely elevated troponin I levels. That can happen due to multiple causes such as fibrin clots, heterophile antibodies, microparticles contained in the sample, rheumatoid factor, interference by bilirubin, hemolysis, and elevated alkaline phosphatase activity. Herein, we present the case of a 66-year-old female who presented with pleuritic chest pain and had a cTnI of 35.5 ng/mL post-transfusion of three units of packed red blood cells. The patient had a complete ischemic workup for ACS, including coronary angiography, which was negative for coronary artery disease.

## Introduction

Cardiac troponins are highly specific and sensitive markers of myocardial cell necrosis and are routinely used in the diagnosis of acute coronary syndrome (ACS). There are several conditions known to cause an elevation in cardiac troponin I (cTnI) levels in the absence of ACS. Frequently reported conditions are acute heart failure, pulmonary embolism, myocarditis, renal failure, sepsis, and severe aortic stenosis [1]. Falsely elevated results can also happen due to analytical interferences with the assay [2,3]. Most common causes include fibrin clots, heterophile antibodies, microparticles contained in the sample, rheumatoid factor, interference by bilirubin, hemolysis, and elevated alkaline phosphatase activity [3,4].

We present a case of a 66-year-old female admitted with atypical chest pain who after receiving three units of packed red blood cells (PRBCs). She has a cTnI level of 35.5 ng/mL (reference range: 0.0-0.04 ng/mL) with negative creatine kinase (CK) biomarkers, absent ST-T wave changes on EKG, normal coronary arteries on coronary angiography, and an unremarkable cardiac MRI. After an extensive workup negative for myocardial ischemia, the elevated cTnI result was deemed likely to be an elevated test result possibly secondary to the transfusion with PRBCs.

This case was presented as an abstract poster at the New York Chapter of the American College of Physicians (NYACP) Annual Scientific Meeting 2013 [5].

## Case presentation

A 66-year-old female with a history of hypertension, diabetes mellitus, and a recent diagnosis of multiple myeloma presented to our facility with sudden pleuritic chest pain. The patient described her pain as midsternal, non-radiating, non-exertional, and exacerbated by inspiration, with no alleviating factors. She denied orthopnea, palpitations, or fever. Vital signs were unremarkable. The physical examination showed mild bibasilar crackles. EKG was read as sinus rhythm with a rate of 84 beats per minute (bpm) with no ST or T wave changes. Pulmonary embolus was ruled out with a CT pulmonary angiography negative for thrombus.

The patient was admitted to the telemetry unit for further monitoring. Cardiac enzymes were drawn, which revealed serial cTnI levels of 0.646 ng/mL, 0.605 ng/mL, and 0.566 ng/mL over 24 hours. CK, CK-MB mass, and index all were negative.

On hospital day 2, the patient underwent transthoracic echocardiography, which showed mild-to-moderately decreased global left ventricular systolic function. Multiple left ventricular regional wall motion abnormalities with a hypokinetic right ventricular free wall and apex were also noted. On hospital day 3, the patient underwent a nuclear perfusion scan, which demonstrated no evidence of ischemia or infarction.

Due to an acute decrease in hemoglobin to 6.7 g/dL, 3 units of PRBCs were transfused and completed on day 3. CTnI levels after PRBC transfusion showed a marked elevation of 35.5 ng/dL. However, CK, CK-MB mass, and index all remained normal. An urgent cardiac catheterization revealed normal coronary arteries. The troponin that night was 32.9 ng/dL, and then it started to go down on the following day to 30.1 ng/dL and 13.2 ng/dL on hospital day 8. Because the patient had a history of multiple myeloma with negative ischemic workup, cardiac amyloidosis was also considered, and the patient underwent a cardiac MRI, which revealed no evidence of infiltrative cardiomyopathy. As the patient continued to be asymptomatic with all workup negative, she was discharged home for outpatient follow-up.

## Discussion

Troponin is a protein complex with three regulatory gene products and is located on the thin filaments of myofibrils. Cardiac troponin C binds calcium, cardiac troponin T attaches to tropomyosin on thin filaments, and cTnI inhibits actomyosin ATPase. Plasma cTnI levels rise and fall with acute myocardial ischemia as a result of their release into plasma either complexed to the contractile apparatus or from free cytosolic troponin pools [6]. Regardless of the mechanism responsible for release into the blood from cardiac myocytes, elevated plasma cTnI levels almost always imply a poor prognosis. Troponin I is the preferred biochemical marker for myocardial damage because of its high myocardial tissue specificity, high sensitivity, and ability to reflect myocardial necrosis in terms of concentration [6]. These circulating troponins are routinely measured in plasma with the use of highly sensitive troponin immunoassays (hsTnI).

The commonly reported assays in the last few decades are the microparticle enzyme immunoassay (MEIA), fluorometric immunoassay, and chemiluminescent immunoassay. The MEIA has an anti cTnI microparticle that binds to circulating troponin, making an antigen-antibody complex. An anti-troponin I alkaline phosphatase conjugate is then added, which binds to the antigen-antibody complex. Finally, 4-methylumbelliferyl is added, which then produces a fluorescent product proportional to the cTnI level. The chemiluminescent assay is a competitive immunoassay that utilizes a monoclonal troponin antibody. The fluorometric enzyme immunoassay utilizes monoclonal antibodies and alkaline phosphatase [7]. Various methods are used to develop immunoassays, and the “sandwich” immunoassay is one of the most reported assay designs in the past few years [8].

There is a strong correlation between cTnI concentration measured by the chemiluminescent assay and the MEIA method. One consideration is the concentrations of cTnI in individual samples may be about four times more elevated when estimated by the MEIA assay than those estimated by the chemiluminescent assay [9]. Interference with cTnI assays can occur independent of analyte concentration from hemolysis, lipemia, or secondary to the effects of anticoagulants and sample storage. Analyte-dependent interference occurs between constituents in the sample, such as heterophile antibodies, rheumatoid factors, human anti-animal antibodies, and other proteins with one or more reagent antibodies [10]. Heterophile antibodies can bind to and capture the conjugate antibodies, simulating cTnI and producing a falsely elevated troponin result. Using antibodies from two different species, as in the MEIA, might decrease the impact of the heterophilic antibodies [10].

More recent assays incorporate heterophilic blocking antibodies preventing such cross-reactivity. Similarly, rheumatoid factor can also cause interference with the assay resulting in elevated cTnI levels. If suspected, a rheumatoid factor blocking agent can be used [4]. Our patient’s plasma was analyzed using the cTnI assay on Dimensions Vista®, a one-step sandwich chemiluminescent immunoassay based on the Luminescent Oxygen Channeling Immunoassay (LOCI) technology. The assay has been designed to decrease interference from endogenous analytes, heterophile antibodies, and rheumatoid factor. A study to confirm the analytical performance of the Dimension Vista LOCI troponin assay showed acceptable performance for diagnosis and risk stratification of patients with ACS [11].

In the United States, more than 20 million patients with suspected ACS present to the emergency departments each year, but most of them do not have myocardial infarction [12]. High-sensitivity cardiac troponin assays allow myocardial infarction to be ruled out earlier. Interpretation of each assay on which cardiac troponin levels are measured requires its own evidence-based method of trending relative changes in cardiac troponin concentrations. The absolute change (delta; δ) criterion uses a calculation designed for use with hsTnI assays and applies the percent difference between two troponin results. A rapid rise (or fall) in the context of timing is more suggestive of ACS than a smaller rise or fall. Reichlin et al. observed that a criterion of an absolute δ over a 2-hour period of >7 ng/L significantly improved the diagnostic specificity for ACS from 93% to 95% [13,14].

The European Society of Cardiology (ESC) recommends using the rapid “rule-in” and “rule-out” algorithms (0 h/1 h algorithm or the 0 h/2 h algorithm). Myocardial infarction can be safely ruled out with a detectable 0-hour high-sensitivity cardiac troponin T (hs-cTnT) level of <12 ng/L, along with an absolute 1-hour change of <3 ng/L [15]. Anand et al. performed a stepped-wedge cluster randomized controlled trial to investigate whether an early rule-out pathway is sufficient and safe for patients with suspected ACS. The study included 31,492 patients with troponin concentrations < 99th percentile at presentation, and it showed 3.3 hours reduction in the length of stay and a 59% decrease in hospital admissions by implementing an early rule-out pathway [16]. In another prospective single-center cohort study, a total of 10,315 patients presenting with suspected ACS were included. Applying an early rule-out pathway using hs-cTnT concentrations <5 ng/L at presentation showed a reduction in the length of stay in the hospital without affecting safety [17].

Hypothesized causes of elevated cTnI levels, unrelated to analytical interference, include transient ischemia, myopericarditis, fluid overload following transfusion, diastolic dysfunction/failure, and small vessel disease not detected on cardiac catheterization. Diastolic dysfunction can lead to occult subendocardial ischemia and excessive wall tension or "myocardial strain" due to the increase in oxygen demand caused by increased muscle mass, along with the decrease in flow reserved because of coronary microcirculation remodeling [18]. The absence of an acute primary coronary thrombotic event caused by arrhythmias, anemia, coronary artery spasm, coronary embolism, hypertension, or hypotension should also be considered [19].

Myocarditis shows two different temporal patterns of cTnI release generally related to the severity of the disease. The most frequent pattern causes a mild increase of serum cTnI (≤0.4 ng/mL) without elevated CK-MB that lasts for less than 72 hours. Higher values of cTnI (≥1.5 ng/mL) can be observed along with elevated CK-MB, wall motion abnormalities in echocardiography, and a prolonged pattern similar to that seen in acute myocardial infarction. Despite different temporal patterns, a similar rate of complications in patients with a positive or a negative cTnI test is seen in myopericarditis. Our patient’s clinical picture and pericardial effusion displayed a pattern of cTnI elevation similar to that seen in myocarditis. Although not segmental, echocardiography showed mild-to-moderate left ventricular dysfunction; however, the delayed enhancement MRI imaging showed no hyperenhancement [20,21].

## Conclusions

An elevated cTnI result should remind the clinician that although troponin immunoassays play an important part in the diagnosis of ACS, other diagnostic modalities including a careful clinical history and physical examination are essential to make this diagnosis. Identification and recognition of non-ischemic/non-ACS related causes of elevated cTnI levels and falsely elevated cTnI results in a patient without ACS can limit unnecessary diagnostic procedures and invasive therapeutic measures and therefore allow for optimal management of these patients.
